# The Importance of Sound Temporary Teeth to Facial Growth and Development

**Published:** 1915-11

**Authors:** J. Lowe Young

**Affiliations:** New York, N. Y.


### THE IMPORTANCE OF SOUND TEMPORARY TEETH TO FACIAL GROWTH AND DEVELOPMENT.

BY J. LOWE YOUNG, D.D.S., NEW YORK, N. Y.

It is assumed that every one will concede that there is a definite relation which the teeth of each dental arch should bear to one another and also to the teeth of the opposing arch—a relation known as normal occlusion. This does not imply that every set of human teeth in this relation is identically the same. The development of these normal conditions necessitates pronounced changes in the organism.

The deciduous denture comprises twenty teeth, ten in each arch. Like the permanent teeth, they should stand in a definite relation to each other and to those of the opposing arch. Their complete eruption extends over a period of from one and a half to two and a half years. When complete, they should be in contact with each other.

It will be observed that a pronounced facial change takes place during this period; the baby's face has been lengthened and carried forward so as to assume the beautiful, pleasing face of the child. No doubt we all have noted many cases where children of our acquaintance at this stage of development were very pleasing in appearance; but later on they became quite "plain-looking" if not homely, which in my opinion is due to lack of normal development of the dental arches.

From the completion of the deciduous denture until the appearance of the first permanent tooth there is a lapse of from three to four years. This does not imply that there is no change in the deciduous denture during this period. If normal development continues, the dental arches expand, and spaces are observed between the incisors. These spaces have never been observed to appear in cases where the deciduous molars have not been used in a vigorous manner. This also causes a facial change, resulting in a broadening of the face and an increase in the size of the mouth.

Unfortunately all children do not proceed along these lines, but I think it safe to state that the percentage of malocclusion of the deciduous dentures at three years of age is very small in comparison to that of permanent dentures.

Between the fifth and eighth years, the first permanent molars, the corner-stone of the teeth, should appear. There are four of these, one on each side of each dental arch, directly back of the last deciduous molars. Owing to the fact that the laity assumes that the child should erupt his first permanent tooth in front, as in the deciduous set, thousands of these teeth are neglected until the fissure cavities have become gaping caves, frequently with exposed pulps, which in the majority of cases means that they will sooner or later be condemned to the forceps.

These first molars are almost always found to be in actual contact with the distal surfaces of the second deciduous molars, and during their eruption there is a further forward movement of the dental arches, and likewise a lengthening of the face.

From the above it will be observed that it is most important that the deciduous teeth should be so cared for that their function shall not be interfered with in the least; also that cavities in their approximal surfaces should be filled so as to maintain their entire mesiodistal diameters, and above all that these teeth shall not be lost prematurely.

When deciduous molars are prematurely lost, it is very advisable that the space should be maintained by some mechanical device. This is particularly necessary if the loss has occurred prior to the normal locking of the first permanent molars.—*Cosmos.*

